# Predictive single nucleotide polymorphism markers for acute oral mucositis in patients with nasopharyngeal carcinoma treated with radiotherapy

**DOI:** 10.18632/oncotarget.18450

**Published:** 2017-06-13

**Authors:** Ziyu Le, Xiaoshuang Niu, Ying Chen, Xiaomin Ou, Guoqi Zhao, Qi Liu, Wenzhi Tu, Chaosu Hu, Lin Kong, Yong Liu

**Affiliations:** ^1^ Department of Radiation Oncology, Fudan University Shanghai Cancer Center, Fudan University, Shanghai 200032, P. R. China; ^2^ Cancer Research Institute, Fudan University Shanghai Cancer Center, Fudan University, Shanghai 200032, P. R. China; ^3^ Department of Oncology, Shanghai Medical College, Fudan University, Shanghai 200032, P. R. China; ^4^ Department of Radiation Oncology, Shanghai General Hospital, Shanghai Jiao Tong University, Shanghai 201620, P. R. China

**Keywords:** genetic polymorphism, nasopharyngeal carcinoma, radiotherapy, oral mucositis, ZNF24

## Abstract

The aim of this study was to investigate the association between the susceptibility of severe oral mucositis (OM) in Chinese nasopharyngeal carcinoma (NPC) patients treated with radiotherapy and single nucleotide polymorphisms (SNPs) across the whole genome. SNPs were screened in a total of 24 patients with NPC and an additional 6 were subjected to mRNA expression analysis. Patients were subdivided into CTC 0-2 (CTC toxicity grade 0, 1, and 2) and CTC 3+ (CTC toxicity grade 3 and above) groups according to their CTC (common toxicity criteria) scores. The GTEx dataset was used to performed eQTL analyses and *in-vitro* functional assays were performed for eQTL-associated genes. Our data identified 7 functional SNPs associated with the development of OM. We observed that rs11081899-A, located in the 5′-UTR of the ZNF24 gene, was significantly correlated with a higher risk of severe mucositis (OR = 14.631, 95% CI = 2.61-105.46, *p* = 1.2 × 10^−4^), and positively associated with *ZNF24* mRNA expression (*p* = 4.1 × 10^−6^) from GTEx dataset. In addition, high *ZNF24* mRNA expression was associated with severe OM in patients with NPC (*p* = 0.02). Further functional assays revealed that ZNF24 knockdown reduced p65 expression and suppressed TNF-α-induced NF-κB activation and pro-inflammatory cytokines release. These findings suggested that rs11081899-A may be a genetic susceptibility factor for radiation-induced OM in patients with NPC, although its value in clinical application needs to be further verified in a large cohort. Also, we suggested that downregulation of ZNF24 may attenuate the development of mucositis by suppressing NF-κB activation.

## INTRODUCTION

Nasopharyngeal carcinoma (NPC) is a common head and neck malignancy in southern China [1]. Radiotherapy alone or in combination with chemotherapy constitutes the standard treatment for patients with NPC. Studies by independent groups have demonstrated excellent local control above 90% and 5-year overall survival of 77-85%, even for advanced stages [2–5]. However, oral mucositis (OM) represents one of the most debilitating and troublesome adverse effect of radiotherapy frequently encountered in patients. It is estimated that approximately 20%-30% of patients receiving standard radiotherapy alone and at least 40%-60% of patients receiving chemoradiotherapy will develop severe OM [6–10]. Swallowing difficulty and local pain caused by mucositis often impair patients’ quality of life. Severe OM may lead to dose reductions and unplanned delays or interruptions of treatment, which have a negative impact on tumor control. In addition, ulcerative mucositis may increase the risk of systemic infection owing to disrupted mucosal barriers, particularly in patients with neutropenia [11].

Patients with NPC treated with radiotherapy experience large variation in the development of OM and most pharmacological interventions for radiation-induced mucositis are unsatisfactory. Although some studies have suggested the effective relief of acute mucositis with palifermin (a recombinant human keratinocyte growth factor) and doxepin rinse (a tricyclic antidepressant), no treatments have been accepted as a standard therapy to date [5, 12–16]. Therefore, the ability to identify specific risk factors and predict the patient risk of developing severe OM prior to cancer therapy represents a recognized goal in the field. Taking such variability into account in the treatment planning phase will allow the radiation therapy to be more individualized. In recent years, a growing consensus considers that, in addition to therapy-related (e.g., fractionation schedule, irradiated volume of normal tissues) and patient-specific (e.g., age, smoking habit) factors, a substantial component of the variation in patient response may be due to genetic susceptibility [17–19].

Single nucleotide polymorphism (SNP), usually defined as polymorphisms in which the minor variant (allele) is present in at least 1% of a given population, accounts for most of the known genetic variation between individuals [20]. At present, only a limited number of studies have evaluated the associations between various genetic SNPs and the risk of radiation-induced OM in NPC patients [20–26]. Most of them were reliant on a candidate gene approach and addressed genes involved in DNA damage response [23]. Although many different associations have been identified, robust replication of the results has only occurred in a very few instances. In addition, as mucositis develops as a consequence of a sequence of related and interacting biologic events [27], restricting the investigated sequence alterations to “DNA damage response” might cause some new and significant candidate SNP markers to be overlooked.

Here, we performed genome-wide screening to identify the SNP markers associated with radiation-induced OM, explored the biological relevance of promising SNPs using expression quantitative trait loci (eQTL) analyses, and further evaluated the function of eQTL-associated genes using functional assays.

## RESULTS

### Subject characteristics

The study population was composed of 30 NPC patients (23 men, 7 women) with a median age of 47.97 years (ranging from 18-70 years) at the time of diagnosis. All patients underwent radical radiotherapy. In addition, 24 patients (80.0%) received chemotherapy; among these, 5 received induction chemotherapy and 19 received induction chemotherapy and adjuvant concurrent chemoradiation therapy (CCRT). Acute mucositis induced by radiotherapy developed in all patients; 60% experienced Grade 1 mucositis (*n* = 18), 10% experienced Grade 2 mucositis (*n* = 3), and Grade 3 accounted for 30% (*n* = 9). No Grade 4 or Grade 5 mucositis was observed.

Our study patients were divided into two cohorts as described in Methods; 24 were involved in the SNP screening study and 6 were subjected to mRNA expression analysis. The distribution of the clinical characteristics for the CTC 0-2 and CTC 3+ group in each study is shown in Tables 1 and 2, respectively. In general, no statistically significant differences were found between the groups in terms of gender, age, tumor stage, or treatment. Based on these results, neither parameter was included in further analyses. Furthermore, no statistically significant differences were found in clinical characteristics between the genotyping and mRNA expression groups (Supplementary Table 1).

**Table 1:** Distribution of clinical characteristics for the CTC 0-2 and CTC 3+ groups in SNP screening

Clinical feature	CTC 0-2 (*n* = 18)	CTC 3+ (*n* = 6)	*p* value
Gender			0.28
Male	13	6	
Female	5	0	
Age, mean ± SD, y	47.83 ± 15.68	52.67 ±8.45	0.48
Clinical stage			0.14
I-II	3	3	
III-IV	15	3	
Treatment			1.00
RT alone^†^	4	1	
RT + CT*	14	5	

† Patients with NPC and severe comorbidities that could not tolerate chemotherapy received radiotherapy alone.

* RT+CT includes induction CT + RT and induction CT + CCRT.

RT = radiotherapy; CT = chemotherapy; CCRT = concurrent chemotherapy.

**Table 2:** General characteristics of patients for mRNA expression analysis

Clinical feature	CTC 0-2 (*n* = 3)	CTC 3+ (*n* = 3)	*p* value
Gender			1.00
Male	2	2	
Female	1	1	
Age, mean ± SD, y	44 ± 11.14	43.33 ±6.03	0.93
Clinical stage			1.00
I-II	1	0	
III-IV	2	3	
Treatment			1.00
RT alone	1	0	
RT + CT*	2	3	

* RT+CT includes induction CT + RT and induction CT + CCRT.

### Genome-wide screening of predictive SNPs

Among the 900,007 SNPs analyzed in the genome-wide screening, 379 showed significant differences in allele frequencies between CTC 0-2 and CTC 3+ groups (*p* < 0.05, Supplementary Table 2). The SNPinfo Web Server (http://snpinfo.niehs.nih.gov/) was utilized to filter these SNPs. We focused on potentially functional SNPs that were located at the 5′-UTR or 3′-UTR of annotated genes. Finally, 7 candidate sites were selected, of which 4 were positioned in the 3′-UTR region of genes and 3 were located in the 5′-UTR of genes. The allele frequency and genotype distribution of the selected SNPs are shown in Table 3.

**Table 3:** Allelic and genotypic associations between candidate SNPs and radiation-induced severe oral mucositis

SNP ID	Group	Allele frequency *		OR	95% CI	Fisher's p	Pearson's p		Genotype frequency **		OR	95% CI	Fisher's p	Pearson's p
rs2044814		G	A						A/G	A/A				
	CTC3+	5(0.417)	7(0.583)	22.660	2.111-1204.335	**4.2300E-04**	**4.2200E-04**		5(0.833)	1(0.167)	2.177	1.674-2.830	**1.40E-04**	**1.40E-04**
	CTC0-2	1(0.028)	35(0.972)						1(0.056)	17(0.944)				
rs1057990		G	A						A/G	A/A				
	CTC3+	4(0.333)	8(0.667)	Inf	2.357-Inf	**3.0000E-04**	**2.9900E-04**		4(0.667)	2(0.333)	2.460	1.809-3.344	**1.50E-04**	**1.49E-04**
	CTC0-2	0(0.000)	36(1.000)						0(0.000)	18(1.000)				
rs1057991		A	C						A/C	C/C				
	CTC3+	4(0.333)	8(0.667)	Inf	2.357-Inf	**3.0000E-04**	**2.9900E-04**		4(0.667)	2(0.333)	2.460	1.809-3.344	**1.50E-04**	**1.49E-04**
	CTC0-2	0(0.000)	36(1.000)						0(0.000)	18(1.000)				
rs1064753		G	A						A/G	A/A				
	CTC3+	4(0.333)	8(0.667)	Inf	2.357-Inf	**3.0000E-04**	**2.9900E-04**		4(0.667)	2(0.333)	2.460	1.809-3.344	**1.50E-04**	**1.49E-04**
	CTC0-2	0(0.000)	36(1.000)						0(0.000)	18(1.000)				
rs11081899		A	G					A/A	A/G	G/G				
	CTC3+	8(0.667)	4(0.333)	14.631	2.609-105.464	**1.2000E-04**	**1.2000E-04**	3(0.500)	2(0.333)	1(0.167)	1.517	1.258-1.830	**2.61E-03**	**2.59E-03**
	CTC0-2	4(0.111)	32(0.889)					0(0.000)	4(0.222)	14(0.778)				
rs281766		C	A					C/C	A/C	A/A				
	CTC3+	4(0.333)	8(0.667)	0.107	0.017-0.545	**9.7300E-04**	**9.7200E-04**	0(0.000)	4(0.667)	2(0.333)	0.633	0.514-0.779	**3.72E-03**	**3.70E-03**
	CTC0-2	30(0.833)	6(0.167)					12(0.667)	6(0.333)	0(0.000)				
rs971232		A	G					A/A	A/G	G/G				
	CTC3+	5(0.417)	7(0.583)	0.122	0.020-0.640	**2.0870E-03**	**2.0840E-03**	1(0.167)	3(0.500)	2(0.333)	0.670	0.535-0.839	**1.09E-02**	**1.08E-02**
	CTC0-2	31(0.861)	5(0.139)					13(0.722)	5(0.278)	0(0.000)				

Bold values are statistically significant

* Alternate allele/reference allele

** Alternate homozygote/heterozygote/reference homozygote. The first four SNPs did not show alternate homozygotes owing to the relatively small sample size.

### Functional annotation of selected SNPs

To further prioritize the candidate SNPs, we annotated transcription factor binding or enhancer elements of these 7 SNPs using HaploReg. As shown in Table 4, rs11081899, which was positioned in the 5′-UTR of the *ZNF24* gene, appeared the most likely to represent a functional SNP. This SNP was located in a region that contained promoter histone marks and DNase hypersensitivity sites in multiple tissues types, and, based on ChIP-Seq experiments data from HaploReg, was found to bind up to 35 transcription factors in multiple tissues. These results support the likelihood that rs11081899 is located within a region with transcriptional regulatory function and may affect transcription regulation via these regulatory elements.

**Table 4:** Summary of the functional annotation for candidate SNPs*

variant	Ref	Alt	ASN freq	Promoter histone marks	Enhancer histone marks	DNAse	Proteins bound	Motifs changed	GENCODE genes	dbSNP func annot	RegPotential	Conservation
rs2044814	A	G	0.24					6 altered motifs	TMEM68	5′-UTR	6.63E-04	1
rs1057990	T	C	0.09					4 altered motifs	WARS2	3′-UTR	0	1.00E-03
rs1057991	C	A	0.09					Mtf1	WARS2	3′-UTR	0	1.00E-03
rs1064753	T	C	0.17				POL24H8	6 altered motifs	ZNF24	3′-UTR	0.14	0.89
rs11081899	G	A	0.4	23 tissues		53 tissues	35 bound proteins	11 altered motifs	ZNF24	5′-UTR	0.3	0.94
rs281766	T	G	0.78	24 tissues		53 tissues	15 bound proteins	7 altered motifs	C2orf47	5′-UTR	0.37	0.3
rs971232	C	T	0.79	IPSC	ESC, IPSC, BRN			6 altered motifs	C2orf69	3′-UTR	0.01	0.01

* Functional annotation based on HaploReg v4.1 and SNPinfo.

**Figure 1:**
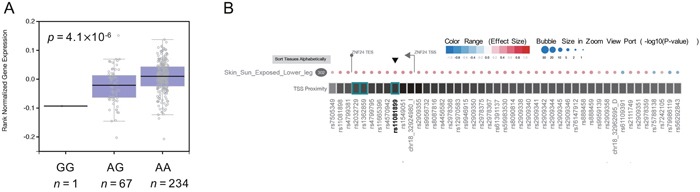
eQTL analysis of rs11081899 **(A)** Boxplots from GTEx data showing the effect of rs11081899 genotypes on *ZNF24* expression in skin-sun-exposed tissue. Numbers below each boxplot indicate the sample size of each genotype. **(B)** Gene eQTL Visualizer plot showing significant eQTLs for *ZNF24* in skin-sun-exposed tissue. An eQTL appears as a circle and a rectangle box on the heat map. The color and size of the circle represent the effect size and *p*-value of the eQTL results, respectively. Additionally, the closer a SNP is to the transcription start site (TSS), the darker the box is. Numbers in black ovals indicate sample sizes. The TSS is drawn as a flag and the direction of the flag indicates the transcription direction.

Accordingly, we queried this SNP for alteration in gene expression in the GTEx dataset and found that rs11081899-A was associated with increased mRNA expression of *ZNF24* (*p* = 4.1 × 10^−6^, Figure 1A) in skin (sun-exposed) tissue. The gene eQTL Visualizer plot is shown in Figure 1B. However, this SNP was not identified as an eQTL for *ZNF24* in other tissues.

### Association between *ZNF24* mRNA expression and the development of OM

**Figure 2:**
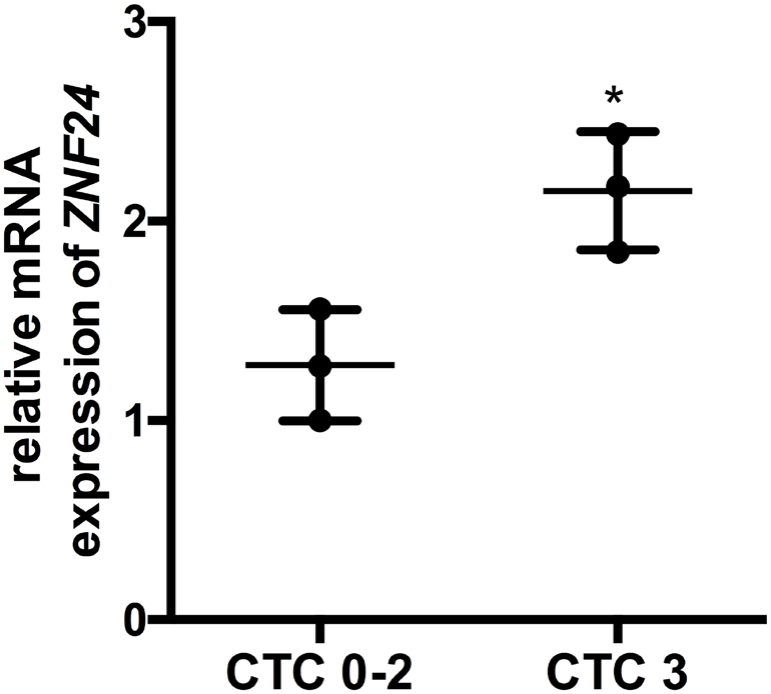
Association between *ZNF24* mRNA expression and severe oral mucositis The relative *ZNF24* mRNA level of peripheral blood lymphocytes in 6 patients with NPC (3 from the CTC 0-2 group, 3 from the CTC 3+ group) was examined by RT-qPCR. *GAPDH* was selected as the internal control. *p < 0.05.

As rs11081899-A was significantly associated with an enhanced risk of severe mucositis (OR = 14.631, 95% CI = 2.61-105.46, *p* = 1.2 × 10^−4^), and positively associated with *ZNF24* mRNA expression (*p* = 4.1 × 10^−6^), we hypothesized that ZNF24 may play a role in the development of radiation-induced mucositis. First, we examined *ZNF24* mRNA expression prior to treatment in 3 sets of paired CTC 0-2 and CTC 3+ patients with NPC using RT-qPCR. As no significant differences were found in the clinical characteristics between these two groups (Table 2), no other parameters were involved in the analysis. The result shown in Figure 2 revealed that *ZNF24* expression prior to treatment was significantly higher in the CTC 3+ group compared with that in the CTC 0-2 group (*p* = 0.02, Figure 2).

### Effect of ZNF24 on TNF-induced NF-κB activation

**Figure 3:**
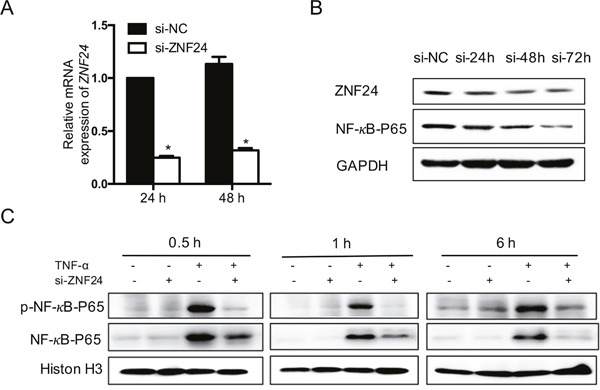
Effect of ZNF24 on NF-κB **(A)** RT-qPCR analysis of *ZNF24* mRNA expression level after siRNA transfection treatment in GM0639 cells for 24 and 48 h. Nontargeting siRNA (si-NC) was used as a control. Data is represented as mean ± SD. **(B)** ZNF24 knockdown reduced p65 expression as determined by western blot analysis. Nontargeting siRNA treatment was used as a control. Protein loading was evaluated by β-actin. **(C)** Downregulation of ZNF24 markedly inhibited TNF-α-induced NF-κB activation. GM0639 cells were treated with siRNA transfection for 48 h and then with TNF-α (1 nM) for the indicated times. Nuclear extracts were prepared and analyzed by western blot using antibodies against p65 and phospho-specific p65 (Ser 536). Nontargeting siRNA treatment was used as a control. Histon-H3 was detected as the loading control. Each assay was performed in triplicate. *p < 0.001.

We next performed functional experiments using human fibroblast GM0639 cells to investigate the potential effect of ZNF24 on OM. Small interfering RNA (siRNA) was transfected into fibroblasts to knockdown ZNF24. The knockdown effect was confirmed by RT-qPCR (Figure 3A). We detected that the level of NF-κB p65 (RelA) was reduced after ZNF24 knockdown (Figure 3B). As NF-κB is known to play an important role in the development of mucositis [25], this finding prompted us to determine whether downregulation of ZNF24 may suppress NF-κB activation in fibroblasts. We treated GM0639 cells with siRNA transfection for 48 h and then with TNF-α (0 or 1 nM) for different durations. Western blot analysis showed that TNF-induced p65 phosphorylation (Ser 563) and nuclear translocation were strongly inhibited by ZNF24 knockdown (Figure 3C).

### Effect of ZNF24 on TNF-α induced pro-inflammatory cytokines

**Figure 4:**
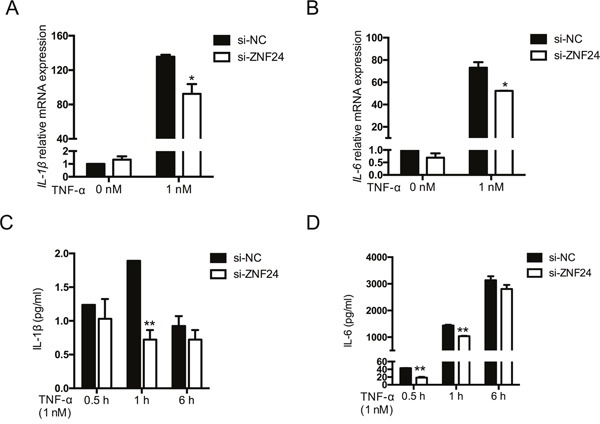
Effect of ZNF24 on TNF-α-induced IL-6 and IL-1β expression GM0639 cells were treated with TNF-α (0, 1 nM) for the indicated times after siRNA treatment for 48 h. **(A, B)** After TNF-α treatment for 1 h, cells were collected and total RNA was extracted. *IL6* and *IL1B* mRNA expression was detected by RT-qPCR. **(C, D)** Cell supernatants were collected at 0.5, 1, and 6 h after TNF-α treatment and the levels of IL-6 and IL-1β secretion were detected by ELISA. Each assay was performed in triplicate. Data was represented as mean ± SD. *p < 0.05, **p < 0.01.

Considering that NF-κB constitutes an important regulator of pro-inflammatory cytokines, we further examined the effect of ZNF24 on IL-6 and IL-1β following TNF-α treatment. GM0639 cells were treated as above, then subjected to RT-qPCR (Figure 4A, 4B) to detect mRNA expression of IL-6 and IL-1β. ELISA (Figure 4C, 4D) was used to determine the level of IL-6 and IL-1β secretion in cultured cell supernatants. As expected, IL-6 and IL-1β were markedly upregulated after TNF-α treatment; furthermore, ZNF24 knockdown attenuated this trend.

## DISCUSSION

Severe OM induced by cancer therapy not only affects the patients’ quality of life, but also treatment of the cancer. Patients with NPC who are treated with radiotherapy alone or in combination with chemotherapy experience large individual differences in the development of severe OM. Predicting this individual risk prior to radiotherapy would likely benefit the personalization and optimization of cancer treatment strategies. Recent studies revealed genetic factors may play a dominant role in determining mucositis risk. In general, for patients receiving systemic therapies, genetic determinants of mucositis risk include genes associated with biological pathways that drive mucositis and genes associated with chemotherapy drug metabolisms. Apparently, the former is more common, compared to enzyme deficiencies [28, 29]. To date, several studies have been performed to investigate the association between SNPs and radiation-induced OM in head and neck cancer(HNC) patients. Most of them evaluated the SNPs in selected candidate genes related to DNA damage and repair. For example, Pratesi et al. [21] assessed the association between acute reactions in HNC patients after radiotherapy and five SNPs in *XRCC1* and *RAD51* genes. Venkatesh GH et al. [25] focused on the polymorphisms in radio-responsive genes. Ren et al. [22] reported that the Ku70 c.1781G>T polymorphism may be a susceptibility factor for severe OM in NPC patients. However, functional assays and robust replication of these results has only occurred in a very few instances.

In addition, clonogenic cell death induced by initial direct DNA damage accounts only for a small number of injured cells and is not of sufficient magnitude to result in clinical mucosal injury. Mucositis develops as a consequence of a cascade of critical biologic mechanisms, such as reactive oxygen species (ROS) generation, NF-κB activation, pro-inflammatory cytokines release and the ceramide pathway activation [28–31]. Brzozowska A et al. [26] demonstrated that SNP of TNFRSF1A (rs4149570) may have association with severe OM in HNC patients. This suggested that, in addition to DNA damage, other pathological variants in the process may also affect the development of OM. Thus, compared to investigating associations in a limited number of SNPs, expanding the scope of screening may help to find more meaningful sites. Accordingly, in the current study we screened predictive SNPs at the genome-wide level. Our data identified 379 SNPs that were significantly associated with severe OM. Among these, 7 were located in the 5′-UTR or 3′-UTR sequences of genes and may thereby have an important role in regulating gene expression, translational efficiency, or mRNA stability [32]. We focused on a SNP termed rs11081899, of which the A allele was significantly correlated with a higher risk of severe OM (OR = 14.631, 95% CI = 2.61-105.46, *p* = 1.2 × 10^−4^). This SNP was positioned in the 5′-UTR of the *ZNF24* gene and was predicted to be within a region with transcriptional regulatory function by HaploReg and SNPinfo.

Zinc finger protein 24 (ZNF24, also termed ZNF191 or KOX17) is a vital zinc finger transcription factor that contains a DNA binding domain and, is indispensable for mammalian embryonic development [33]. Recent studies have found that ZNF24 is a pleiotropic factor involved in many critical biologic events and pathways, such as tumor progression and angiogenesis, brain development, DNA replication, and DNA damage response [34–39]. We queried rs11081899 in the GTEx dataset and confirmed that a transcriptional regulatory function exists at this risk locus for the *ZNF24* gene in the skin (sun exposed) tissue. In particular, rs11081899-A was associated with increased mRNA expression of *ZNF24* (*p* = 4.1 × 10^−6^). Additionally, our data revealed that high *ZNF24* mRNA expression was associated with severe OM in patients with NPC (*p* = 0.02). These findings suggested that ZNF24 may play a role in the development of mucositis.

NF-κB is also known to play a critical role in the development of OM [40]. This inducible transcription factor is typically identified as a dimeric complex composed of different members of the Rel/NF-κB family of polypeptides. The p65/p50 heterodimer is considered to be the classic form that resides in the cytoplasm in resting status, whereas upon activation the heterodimer is translocated to the nucleus where it binds to specific response elements in the DNA [41, 42]. NF-κB targets a variety of genes associated with apoptosis and the expression of cytokines, chemokines, stress responders and cell adhesion molecules, most of which are of considerable importance in the pathogenesis of mucosal injury [40, 43, 44]. In the initiation stage of OM, NF-κB can be activated directly by radiation or by the ROS that are generated by X-rays or chemotherapy. Upon activation, NF-κB can modulate the pro-inflammatory cytokines and apoptosis genes in the BCL family. NF-κB can also activate COX-2 to produce prostaglandins. In addition, TNF-α can positively feedback on NF-κB and thus upregulate and amplify the injury message [27–29]. In the current study, we found that ZNF24 knockdown significantly reduced p65 subunit expression and markedly suppressed TNF-α induced NF-κB activation in fibroblasts. These observations imply that downregulation of ZNF24 may attenuate the development of OM by suppressing NF-κB activation.

Pro-inflammatory cytokines regulated by NF-κB, such as IL-6, IL-1β and TNF-α, account for an important mechanistic component in the pathogenesis of mucositis [28]. The protein levels of these cytokines in both tissue and peripheral blood are positively associated with the severity of mucosal toxicity [27, 45–47]. To further investigate this relationship, we examined the effect of ZNF24 on pro-inflammatory cytokines. A study from Fei et al. [48] has reported that knockdown of the mouse zinc finger protein Zfp 191 could reduce inflammatory cytokine levels during LPS stimulation. Similarly, we found that ZNF24 knockdown attenuated the upregulation of IL-6 and IL-1β induced by TNF-α treatment. These findings thereby support the hypothesis that ZNF24 impacts the development of OM via NF-κB and pro-inflammatory cytokines.

In summary, our data provide preliminary evidence that rs11081899 may serve as a genetic susceptibility factor for radiation-induced severe OM in Chinese NPC patients and that ZNF24 may affect the development of this complication by regulating NF-κB and pro-inflammatory cytokines. To our knowledge, our research represents the first attempt to analyze the effect of genetic variants across the whole genome on radiation-induced OM. In addition, this is also the first report that demonstrates the impact of ZNF24 on NF-κB. However, the population in this study was relatively small, impairing the statistical power of the results. Additionally, we identified a transcriptional regulatory function of rs11081899 on ZNF24 and demonstrated the effect of ZNF24 on NF-κB expression; however, further studies to investigate this phenomenon in more detail were not performed. Moreover, we used skin fibroblasts in functional assays, and it may be more convincing to use human gingival fibroblasts or oral keratinocytes. Large sample studies and in-depth functional experiments are needed to confirm our results. Validation of our findings would support the utility of rs11081899 genotyping status in the design of individual treatment regimens for patients with NPC.

## MATERIALS AND METHODS

### Study patients and treatments

From December 2011 to February 2013, 30 Chinese patients with NPC that fulfilled the following criteria were enrolled in this study: pathologically confirmed NPC, previously untreated, no evidence of distant metastasis, no previous radiotherapy and/or chemotherapy, receiving whole course of radical radiotherapy in our institution, available for correct follow-up. There was no restriction on gender or age. All patients provided written informed consent. Ethical approval was granted from the ethics committee of Fudan University Shanghai Cancer Center.

Blood samples were collected prior to starting treatment and stored at −80°C until analysis. All patients underwent radiotherapy. The mean tumor dose was 69.4 Gy (range 66-70.4 Gy) in 30-35 fractions. Radiation was delivered using a simultaneous integrated boost-intensity modulated-radiation therapy (IMRT) technique. All patients were treated with one fraction daily, for five days per week. Tumor, lymph node, and metastasis (TNM) classification was established as per to the staging system of the 7th edition of the American Joint Committee of Cancer (AJCC). Cisplatin-based chemotherapy was recommended to medically fit patients with stage II-IV disease. In general, 80% of patients received chemotherapy including induction chemotherapy and/or concurrent chemotherapy (CCRT). Chemotherapy regiments were based on derivatives of platinum, 5-fluorouracile, taxanes, and/or nimotuzumab.

### Mucositis evaluation

Oral mucositis caused by anticancer treatments was documented and evaluated according to the National Cancer Institute Common Terminology Criteria for Adverse Events 3.0 version (CTCAE 3.0) [48] weekly from the beginning of radiotherapy to one month after completion of treatment. The highest grade of oral mucositis that patients experienced during follow-up was recorded as their common toxicity criteria (CTC) scores. Patients were subdivided into CTC 0-2 (CTC toxicity grade 0, 1, and 2) and CTC 3+ (CTC toxicity grade 3 and above) groups according to their CTC scores. The mildly affected (CTC 0-2) group was regarded as the reference group.

The CTCAE v3.0 toxicity grades for acute OM are as follows: Grade 0, no mucositis; Grade 1, erythema of the mucosa; Grade 2, patchy ulcerations or pseudomembranes; Grade 3, confluent ulcerations or pseudomembranes; or bleeding with minor trauma; Grade 4, tissue necrosis or significant spontaneous bleeding; life-threatening consequences; and Grade 5, death [49].

### Genotyping assays and functional analysis

After follow-up and grouping, three blood samples from each of the CTC 0-2 and CTC 3+ groups were selected at random to extract RNA for subsequent mRNA expression analysis. RNA extraction is described in detail below. The remainder (24 blood samples) were used to extract genomic DNA for genotyping. Genomic DNA extraction was performed using the TIANamp Blood DNA kit (TIANGEN, Beijing, China) following the manufacturer's instructions. The DNA samples were checked for quality and quantity by DNA electrophoresis and Nanodrop^TM^ 2000 (Thermo Fisher Scientific, Wilmington, DE, USA) prior to genotyping. Each DNA sample was stored at −20°C until analysis.

SNPs were genotyped using the HumanomniZhongHua-8v1 SNP Array (Illumina, San Diego, CA, USA) with 900,015 probes. Of these SNPs, 900,007 coordinates were used in subsequent analyses with the Genome Studio Genotyping Module v1.0 after quality control (sample call rate, ≥98%; SNP call rate, ≥99%; minor allele frequency, ≥1%; Hardy-Weinberg equilibrium (HWE), *p* > 10^−5^). The final genotyping rate was 99.84% among our 24 samples (6 cases and 18 controls).

After genome-wide screening, the screened-out SNPs were filtered using the SNPinfo Web Server (http://snpinfo.niehs.nih.gov/) [50] to selected potentially functional SNPs. In addition, HaploReg v4.1 (http://archive.broadinstitute.org/mammals/haploreg/haploreg.php) [51] was used to examine whether any of the SNPs were annotated as transcription factor binding or enhancer elements. GTEx dataset (Release V6) [52] was used to performed eQTL analyses to examine the effects of SNPs on mRNA expression.

### Cell culture and cytokines

Human fibroblast cells from the skin of an unaffected individual, GM0639 (termed GM cells), were a generous gift from Dr. Lian Xue (Medical College of Soochow University, Suzhou, China). The cells were cultured in Dulbecco's modified Eagle medium (Gibco, Gaithersburg, MD, USA) supplemented with 10% fetal bovine serum (Gibco) at 37°C with 5% CO_2_. Recombinant human TNF-α, purified to homogeneity with a specific activity ≥ 2 × 10^7^ U/mg, was purchased from PeproTech (Rocky Hill, NJ, USA). A 50 nM solution of TNF-α was prepared in a buffer containing 0.1% bovine serum albumin, stored as small aliquots at −80°C, and then thawed and diluted as needed in cell culture medium.

### siRNA transfection

The siRNA against human ZNF24 and control non-targeting siRNA were purchased from Ribobio (Guangzhou, China). Transient transfections were performed using Lipofectamine 2000 according to the manufacturer's instructions (Invitrogen, Carlsbad, CA, USA). Cells were analyzed at 24, 48, and 72 h after transfection.

### RNA isolation from cells and real-time RT-PCR

Total RNA from the blood samples were extracted using an RNAprep Pure Hi-Blood kit (TIANGEN). Total RNA from GM0639 cells was isolated using the RNAprep pure cell/bacteria kit (TIANGEN). All procedures were based on the manufacturer's instructions. The concentration of each RNA sample was measured using a NanoDrop^TM^ 2000 Spectrophotometer (Thermo Fisher Scientific). Subsequently, 500 ng RNA was reverse-transcribed into cDNA using the PrimeScript^TM^ RT Reagent kit (TaKaRa, Shiga, Japan) according to the manufacturer's protocol. Quantitative PCR was performed on a LightCycler 480 System (Roche, Basel, Switzerland) in 20 μl reactions by using the LightCycler 480 SYBR Green I Master Mix (Roche). *GAPDH* was selected as the internal control. The primer sequences were as follows: Human *GAPDH* (forward, 5′-TCT CCT CTG ACT TCA ACA GCG AC-3′, reverse, 5′-CCC TGT TGC TGT AGC CAA ATT C-3′), Human *ZNF24* (forward, 5′-CGA GAT CAT CAT CCA GAG AA-3′, reverse, 5′-GTC CTA CCA TCA TCA TCA CA-3′), Human *IL6* (forward, 5′-TGT AGT GAG GAA CAA GCC AGA G-3′, reverse, 5′-TAC ATT TGC CGA AGA GCC-3′), Human *IL1B* (forward, 5′-GAA GCT GAT GGC CCT AAA CA -3′, reverse, 5′- AAG CCC TTG CTG TAG TGG TG-3′). The relative expression levels were calculated using the 2^−Δ Δ Ct^ method [53].

### Western blot

Whole cell lysates were prepared using RIPA lysis buffer (Roche) following the manufacturer's instructions. Nuclear protein was extracted from TNF-α-treated cells using a Nuclear and Cytoplasmic Protein Extraction Kit from Beyotime (Shanghai, China) according to the manufacturer's protocol. The protein concentrations were determined using a Pierce BCA protein assay kit (Thermo Fisher Scientific). An equal amount of each protein extract was separated on 10% sodium dodecyl sulfate-polyacrylamide gels and electrophoretically transferred to polyvinylidene difluoride membranes (Millipore, Billerica, MA, USA). The blotted membrane was blocked with 5% bovine serum albumin for 1 h at room temperature and stained with the specific first antibodies overnight at 4°C. After incubating with horseradish peroxidase-conjugated goat-anti-rabbit or goat-anti-mouse antibodies for 1 h at room temperature, the protein bands were visualized using ECL Blotting Detection Reagents (Thermo Fisher Scientific) with a Chemioscope Mini system (Bioshine, Shanghai, China). The density of each protein band was quantified using Image J 1.51h software (NIH, Bethesda, MD, USA). The primary antibodies used in this study were as follows: anti-ZNF24 rabbit monoclonal (1:4000, Abcam, Cambridge, UK), anti-NF-κB p65 rabbit monoclonal, anti-phospho-NF-κB p65 (Ser536) rabbit monoclonal, anti-Histone H3 mouse monoclonal (1:1000, Cell Signaling Technology, Danvers, MA, USA), and anti-GAPDH mouse monoclonal (1:10000, Proteintech, Chicago, IL, USA).

### ELISA

Cells were cultured in 6-well plates. After siRNA transfection for 48 h, cells were treated with TNF-α (1 nM) for different time periods (0.5, 1, 6 h). The supernatants were collected and the protein levels of IL-6 and IL-1β were detected in duplicate using ELISA kits (Multi Science, Hangzhou, China) according to the manufacturer's instructions.

### Statistical analysis

Differences in the distribution of selected clinical characteristics between patients with NPC in the CTC0-2 and CTC3+ groups were evaluated using a Student's t-test or Chi-square test. HWE analysis was performed using PLINK (version 1.9) [54]. The association between the development of severe acute OM and SNPs was conducted by using PLINK. *p* values < 0.05 were used for the initial screening of candidate SNPs owing to the relatively small sample size. For the selected SNPs, a Fisher-exact test and Chi-square test were used for the allelic and genotypic association between the disease and SNPs via R (version 2.14.0). The hazard ratio (HR) and 95% confidence interval (CI) were also calculated. For functional assays, different groups with various treatments were compared using a Student's t-test. All tests were two-sided and statistical significance was defined at *p* < 0.05.

## SUPPLEMENTARY MATERIALS TABLES





## Abbreviations

OM: oral mucositis
NPC: nasopharyngeal carcinoma
SNP: single nucleotide polymorphism
CTC: common toxicity criteria
eQTL: expression quantitative trait loci
GTEx: Genotype-Tissue Expression project
TNF-α: tumor necrosis factor alpha
IL-1β: interleukin 1β
IL-6: interleukin 6
NF-κB: nuclear factor kappa-light-chain-enhancer of activated B cells
ZNF24: Zinc finger protein 24
ChIP-Seq: chromatin-immunoprecipitation-sequencing
RT-qPCR: quantitative real-time reverse transcription-polymerase chain reaction
OR: odds ratio
CI: confidence interval
