# Supplementary material for: Predictive single nucleotide polymorphism markers for acute oral mucositis in patients with nasopharyngeal carcinoma treated with radiotherapy
Source: Oncotarget. 2017 Jun 13;8(38):63026–37. doi: 10.18632/oncotarget.18450 (PMC5609900; doi:10.18632/oncotarget.18450)
Supplement: Supplementary file 1 [file oncotarget-08-63026-s001.pdf]

Predictive single nucleotide polymorphism markers for acute oral mucositis in patients with nasopharyngeal carcinoma treated with radiotherapy

SUPPLEMENTARY MATERIALS

Supplementary Table 1: Clinical features of patients in genotyping study and mRNA expression study

Clinical feature	genotype group	mRNA expression group	*p* value
Gender			0.60
Male	19	4	
Female	5	2	
Age, mean±SD, y	49.04± 14.21	43.67± 8.02	0.38
Clinical stage			1.00
I-II	6	1	
III–IV	18	5	
Treatment			1.00
RT alone	5	1	
RT +CT	19	5	

Supplementary Table 2: Detailed Information of 379 SNPs associated with severe oral mucositis

See Supplementary File 1
